# Anti‐Inflammatory Activity of *Cotula cinerea* Essential Oil: Integrated Chemical, In Vivo, Histological, and Computational Evaluation

**DOI:** 10.1002/cbdv.71793

**Published:** 2026-10-06

**Authors:** Habiba Haddad, Mohammed Larbi Benamor, Yahia Bekkar, Ouafa Boudebia, Salah Neghmouche Nacer, Elhafnaoui Lanez, Aicha Adaika, Taher M. Abdelhameed, Lazhar Bechki, Touhami Lanez, Stefania Garzoli

**Affiliations:** ^1^ Faculty of Technology Department of Process Engineering and Petrochemistry Laboratory of Valorisation and Technology of Saharian Resources (VTRS) University of El Oued El Oued Algeria; ^2^ Department of Physical Sciences Higher Normal School of Kouba (ENS Kouba) Algiers Algeria; ^3^ Faculty of Exact Sciences Department of Chemistry University of El Oued El Oued Algeria; ^4^ Faculty of Medical Technologies Department of Medical Nutrition University of Zawia Zawia Libya; ^5^ Faculty of Mathematics and Material Sciences Department of Chemistry Laboratory of Valorisation and Promotion of Saharan Resources (VPRS) Kasdi Merbah University Ouargla Algeria; ^6^ Department of Chemistry and Technologies of Drug Sapienza University Rome Italy

**Keywords:** anti‐inflammatory, *Cotula cinerea*, essential oil, GC‐MS, histopathological analysis, MM‐PBSA, molecular docking, molecular dynamics

## Abstract

The anti‐inflammatory activity of *Cotula cinerea* essential oil (EO) was evaluated using integrated chemical, in vivo, histological, and computational approaches. Gas chromatography–mass spectrometry (GC–MS) identified trans‐thujone (51.86%), santolina triene (10.60%), and sabinene (6.17%) as the most abundant constituents. In benzylthiouracil (BTU)‐treated Wistar rats, EO administration reduced the liver and kidney indices from 4.36% to 2.70% and from 0.65% to 0.52%, respectively; red blood cell count increased from 4.90 to 7.17 × 10^12^/L and hemoglobin from 9.40 to 15.40 g/dL. Aspartate aminotransferase decreased from 226.06 to 111.10 U/L and alanine aminotransferase from 116.00 to 71.56 U/L. C‐reactive protein and renal markers also moved toward control values, and histological examination showed improved liver and kidney architecture. Computational analyses were used as hypothesis‐generating complements to the experimental findings. Molecular docking predicted the most favorable cyclooxygenase‐2 (COX‐2) scores for cis‐verbenyl acetate (CVA; −7.01 kcal/mol) and camphor (CAM; −6.66 kcal/mol). Molecular dynamics (MD) trajectories and molecular mechanics/Poisson–Boltzmann surface area (MM‐PBSA) estimates indicated persistent simulated interactions, with COX‐2 binding free energies of −43.67 ± 3.80 and −39.93 ± 2.59 kcal/mol, respectively. Absorption, distribution, metabolism, excretion, and toxicity (ADMET) outputs were favorable for CVA and CAM but remain predictive.

## Graphical abstract

*Cotula cinerea* essential oil was chemically profiled and evaluated in an exploratory in vivo model with histological assessment, while computational analyses generated hypotheses for interactions of selected constituents with COX‐2 and BSA. The predictive findings require direct experimental validation.

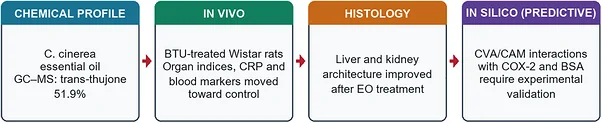

## 1 Introduction

Inflammation is a multifaceted physiological response triggered by tissue injury, infection, or irritation, and is orchestrated through a wide network of immune mediators, signaling pathways, and cellular responses. Acute inflammation represents an essential protective mechanism for host defense and tissue repair. However, when inflammation becomes chronic, it contributes to the pathogenesis of several non‐communicable diseases, including cardiovascular disorders, rheumatoid arthritis, neurodegenerative conditions, and metabolic syndromes such as type 2 diabetes mellitus and obesity [1, 2]. Conventional anti‐inflammatory therapies—most notably non‐steroidal anti‐inflammatory drugs (NSAIDs) and corticosteroids—remain effective but are frequently associated with adverse effects, including gastrointestinal irritation, nephrotoxicity, hepatotoxicity, and immunosuppression. These limitations underscore the urgent need for safer, sustainable, and more biocompatible therapeutic alternatives [3, 4].

Medicinal plants are important sources of structurally diverse bioactive compounds with reported antioxidant, anti‐inflammatory, and immunomodulatory properties [5, 6, 7]. Phytochemicals such as flavonoids, sesquiterpenes, polyphenols, and essential‐oil constituents have been reported to influence inflammatory pathways involving nuclear factor kappa B (NF‐κB), cyclooxygenase‐2 (COX‐2), and lipoxygenase (LOX), as well as mediators such as tumor necrosis factor‐alpha (TNF‐α), interleukin‐6 (IL‐6), and interleukin‐1 beta (IL‐1β) [5, 6].

Among these promising botanicals, *Cotula cinerea* (family: Asteraceae), a perennial herb widely employed in North African folk medicine, has recently garnered considerable scientific interest. Traditionally used in the treatment of gastrointestinal, dermatological, and respiratory ailments, emerging pharmacological evidence highlights its potent anti‐inflammatory and antioxidant activities. These effects are largely attributed to its diverse phytochemical composition, which includes flavonoids, monoterpenes, and sesquiterpene lactones [8, 9, 10]. In vitro and in vivo studies suggest that *C. cinerea* exerts its effects by inhibiting cyclooxygenase (COX) and lipoxygenase (LOX) enzymes, suppressing nitric oxide (NO) production, and mitigating reactive oxygen species (ROS) generation [11, 12, 13, 14].

The present study evaluates the anti‐inflammatory potential of *C. cinerea* EO through chemical profiling, a BTU‐induced rat model, histological assessment, and computational predictions. Cyclooxygenase‐2 (COX‐2) was selected because it catalyzes prostaglandin formation during inflammation, whereas bovine serum albumin (BSA) was included only as a model serum transport protein in the computational analyses. Docking and simulation were used to explore possible ligand–target interactions and generate testable hypotheses; they were not treated as evidence of biological activity or proof of mechanism. This integrated design connects complementary datasets while clearly separating experimental observations from predictive results.

## 2 Materials and Methods

### 2.1 Plant Material

*Cotula cinerea* specimens were collected from a desert forest area in southeastern Algeria, specifically the Hassi Khalifa region of El Oued province. The site is geographically located at latitude 23°09′ N and longitude 48°10′ E, with an elevation of approximately 44 meters above sea level. This region lies within the northern Sahara and is classified as a hyper‐arid desert bioclimatic zone. It is characterized by prolonged drought, intense solar radiation, and scarce vegetation dominated by xerophytic and halophytic plant species [15, 16].

Plant identification was based on morphological features using the North African Flora and verified using the NCBI Taxonomy database (Taxonomy ID: 282208). The identification was confirmed by Prof. [Atef CHOUIKH], Department of Biology, University of El Oued. A voucher specimen was preserved and deposited at the herbarium of the Faculty of Natural Sciences and Life, University of El Oued, under the code CC‐NEGR2023 [17].

### 2.2 Essential Oil Extraction

The essential oil (EO) of *C. cinerea* was extracted by hydrodistillation using a Clevenger‐type apparatus, a standard method for obtaining high yield and purity [18]. A 100 g portion of fresh plant material was accurately weighed, rinsed with distilled water to remove surface impurities, and transferred to a distillation flask containing boiling regulators. Hydrodistillation was carried out at 100°C for 3.5 h.

Following distillation, the EO was separated from the aqueous distillate by liquid–liquid extraction with diethyl ether. The organic phase was dried over anhydrous sodium sulfate to remove residual moisture, then filtered through qualitative filter paper to eliminate particulate matter and solvent traces. The oil was subsequently concentrated under reduced pressure using rotary evaporation to remove excess diethyl ether. The final product was collected in amber glass vials and stored at 5°C in the dark to maintain its chemical integrity and prevent degradation [18, 19].

### 2.3 Gas Chromatography‐Mass Spectrometry Analysis

The chemical composition of *C. cinerea* essential oil (EO) was determined using an HP 5890 Series II gas chromatograph coupled to an HP‐MSD 5972 mass‐selective detector. A 1 µL aliquot was injected in split mode (1:20), with nitrogen (N_2_) as the carrier gas at a constant flow rate of 1 mL/min.

Chromatographic separation was achieved on an HP‐5 MS capillary column (30 m × 0.25 mm i.d., 0.25 µm film thickness). The oven temperature program was as follows: initial temperature of 60°C held for 8 min, ramped at 2°C/min to 250°C and held for 15 min, then increased at 10°C/min to 300°C and maintained for 5 min to ensure elution of high‐boiling‐point constituents. The injector and detector temperatures were set to 250°C and 280°C, respectively.

Mass spectra were acquired by electron‐impact (EI) ionization at 70 eV over m/z 40–500. Constituents were assigned tentatively by agreement of their mass spectra with the NIST 20 and WILEY275 libraries and by comparison of experimentally determined retention indices, obtained with a homologous n‐alkane series, with literature values [18, 20, 21]. One analytical injection was used for the reported composition (*n* = 1 analytical profile). Relative composition was calculated by chromatographic peak‐area normalization without an internal standard or compound‐specific response‐factor correction. The percentages are therefore semi‐quantitative. Calibration curves, linearity, and limits of detection or quantification were not determined because absolute concentrations were not measured. Authentic standards were not used; major constituents remain putatively identified pending standard confirmation.

### 2.4 In Vivo Assessment of Anti‐Inflammatory Potency

#### 2.4.1 Animal Care and Experimental Design

This study employed twenty (20) male Wistar rats, initially weighing 150–200 g, obtained from the Pasteur Institute, Algiers. Animals were housed in the animal facility of the Faculty of Natural and Life Sciences, University of El Oued, under standardized environmental conditions: temperature 25°C ± 2°C, relative humidity 50%–60%, and a 12 h light/12 h dark cycle. Rats were kept in polypropylene cages (five per cage) with sawdust bedding and provided standard chow and water ad libitum. Body weights were recorded regularly to monitor physiological changes during the experimental period, thereby ensuring reliability and minimizing external variables that could influence inflammatory or biochemical markers [22].

All experimental procedures were carried out in accordance with institutional and national ethical guidelines for the care and use of laboratory animals. The study protocol was reviewed and approved by the Scientific Council Committee of the Faculty of Natural and Life Sciences, University of El Oued, Algeria (Approval No. 10/S.C./FL/NS/EU/2024).

#### 2.4.2 Induction of Hepatic Inflammation Using Benzylthiouracil

Experimental hepatitis was induced by administering benzylthiouracil (BTU) at a dose of 2 mg/kg/day for 21 consecutive days. BTU, a thiourea derivative, induces hepatotoxicity through several mechanisms. First, it may act as a hapten by binding to hepatic proteins, thereby eliciting an immune‐mediated hypersensitivity response characterized by increased eosinophil counts and elevated transaminases (ALT, AST). Second, hepatic metabolism of BTU can generate reactive intermediates that exert direct cytotoxic effects and promote oxidative stress. Finally, prolonged administration may provoke drug‐induced autoimmunity against hepatocytes, further exacerbating liver injury [23, 24].

#### 2.4.3 Experimental Groups and Treatment Regimens

After completion of the induction phase, the animals were randomly divided into four experimental groups, each consisting of five rats. The control group received only standard chow, bran, and water ad libitum, while the BTU group was administered benzylthiouracil (2 mg/kg/day) for 21 consecutive days to establish the hepatotoxic model. A third group was treated with BTU in combination with *Cotula cinerea* essential oil (100 µL/day), which was administered orally during the final 7 days of the induction period. The fourth group received BTU together with ibuprofen (2 mg/kg/day) during the same treatment window, serving as the reference drug group. This design enabled a comparative assessment of the therapeutic effects of *C. cinerea* essential oil relative to a conventional anti‐inflammatory agent under conditions of BTU‐induced hepatic inflammation [25, 26, 27].

#### 2.4.4 Sample Collection and Organ Analysis

At the end of the experimental period, the animals were fasted for 16 h, anesthetized with 94% chloroform, and euthanized by decapitation. Blood samples were collected for hematological and biochemical analyses, while the liver and kidneys were carefully excised, weighed, and rinsed with 0.9% NaCl solution to remove residual blood. The harvested tissues were fixed in 10% buffered formalin for subsequent histopathological examination. The organ weight index (OWI) was calculated according to the formula:

$$OWI=\left(\frac{\mathrm{Organ}\mathrm{Weight}\;(g)}{\mathrm{Body}\mathrm{Weight}\;(g)}\right)\times 100$$

### 2.5 In Silico Evaluation of Anti‐Inflammatory Activity

#### 2.5.1 ADMET and Drug‐Likeness Prediction

The pharmacokinetic profiles and drug‐likeness of the major bioactive compounds identified from *C. cinerea* EO were assessed using the SwissADME and pkCSM platforms [28, 29]. These analyses provided insights into absorption, distribution, metabolism, excretion, and toxicity (ADMET) parameters, enabling the identification of molecules with favorable oral bioavailability and pharmacokinetic characteristics relevant to anti‐inflammatory therapy.

#### 2.5.2 Molecular Docking

The three‐dimensional structures of the principal phytoconstituents were retrieved from the PubChem database in SDF format and converted into PDB files using Open Babel [30, 31]. Ligand geometries were further optimized through energy minimization with the MMFF94 force field to obtain stable conformations suitable for docking simulations.

Protein crystal structures, including bovine serum albumin (BSA; PDB ID: 6QS9) and cyclooxygenase‐2 (COX‐2; PDB ID: 1PXX), were obtained from the RCSB Protein Data Bank [32]. Proteins were preprocessed following standard docking protocols [33, 34, 35, 36], which involved removal of crystallographic water molecules and nonessential heteroatoms, followed by conversion into AutoDock‐compatible PDBQT format. Binding interactions and docking poses were subsequently analyzed using LigPlot v2.3 [37].

Molecular docking was performed using AutoDock Tools (version 1.5.7) [38] with the Lamarckian Genetic Algorithm (LGA) to predict optimal binding conformations between ligands and selected target proteins [36, 39, 40]. Prior to docking, both ligands and proteins were converted into PDBQT format, Gasteiger charges were assigned, and torsional degrees of freedom were defined. The docking grid was configured to cover the active site of each protein, ensuring comprehensive sampling of potential binding interactions.

#### 2.5.3 Molecular Dynamic Simulation

Molecular dynamics (MD) simulations were conducted to evaluate the structural stability, conformational dynamics, and interaction persistence of the top‐ranked protein–ligand complexes identified from molecular docking. The best‐scoring docked pose for each target was used as the starting structure. All simulations were performed using GROMACS 2024 on a GPU‐accelerated platform [41].

Protein atoms were parameterized using the CHARMM36 force field, while ligand topologies were generated via the SwissParam server [42]. Each complex was placed in a cubic simulation box with a minimum solute–box distance of 1.0 nm and solvated using the TIP3P explicit water model. The systems were neutralized and adjusted to physiological ionic strength by adding Na^+^ and Cl^−^ ions [21, 35].

Energy minimization was performed using the steepest descent algorithm, followed by conjugate gradient refinement, until the maximum force on any atom was below 10.0 kJ mol^−1^ nm^−1^ [43]. The minimized systems were equilibrated in two successive phases: an NVT ensemble for 100 ps at 300 K using the velocity‐rescaling (v‐rescale) thermostat with a coupling constant of 0.1 ps, followed by an NPT ensemble for 100 ps at 1 bar using the Berendsen barostat with a coupling constant of 2.0 ps [44, 45]. Production MD simulations were then executed for 100 ns under the NPT conditions at 300 K and 1 bar, with trajectories saved every 10 ps for analysis.

Post‐simulation analyses were performed to characterize the dynamic behavior of the complexes. Backbone root mean square deviation (RMSD) was calculated to assess overall structural stability, while root mean square fluctuation (RMSF) quantified residue‐level flexibility. Structural compactness was evaluated using the radius of gyration (*R*_g_), and solvent exposure was assessed through solvent‐accessible surface area (SASA).

#### 2.5.4 MM/PBSA Binding Free Energy

MM/PBSA calculations were performed using the gmx_MMPBSA package [46] to estimate protein–ligand binding free energies. Frames were extracted from the final 10 ns of each equilibrated MD trajectory at 10 ps intervals. Binding free energy (Δ*G*_bind_) was computed as:

(3) $$\Delta G_{\mathrm{Bind}}=G_{\mathrm{complex}}-\left(G_{\mathrm{protein}}+G_{\mathrm{ligand}}\right)$$

where *G_complex_*, *G_protein_*, and *G_ligand_* are the free energies of the complex, unbound protein, and the ligand, respectively. Each term was decomposed into gas‐phase energy (ΔE_Gas_ = ΔE_vdW_ + ΔE_ele_) and solvation free energy (Δ*G*_solv_ = Δ*G*_PB_ + Δ*G*_NPB_). The entropic contribution (–*T*Δ*S*) was omitted, which is standard in comparative MM/PBSA analyses due to high computational cost and minimal effect on relative binding trends [47].

### 2.6 Statistical Analysis

In vivo data are presented as mean ± standard deviation (SD), with five independent animals per group (*n* = 5). No transformation or outlier exclusion was applied. Each endpoint was analyzed independently by ordinary one‐way analysis of variance (ANOVA), followed by Tukey's honestly significant difference test for all pairwise comparisons. Tests were two‐sided, with *α* = 0.05; *p* < 0.05 was considered statistically significant. The ANOVA model assumes independent observations, approximately normal residuals, and similar within‐group variances. The archived dataset contained group means and SDs rather than animal‐level residuals, so formal residual‐normality and variance tests could not be repeated; equal group sizes were retained throughout. During revision, the reported tabled endpoints were independently recalculated from the archived summary statistics using Python 3.12 and SciPy 1.18.1. Molecular‐dynamics and MM‐PBSA values are descriptive trajectory‐frame means ± SD and were not included in the biological group comparisons.

## 3 Results and Discussion

### 3.1 Extraction Yield

Hydrodistillation of the aerial parts of *Cotula cinerea* yielded 0.90% (v/w) of EO. This value is considerably higher than those previously reported by Djellouli et al. [48] and Mekhadmi et al. [49], who recorded yields of 0.282% and 0.45% (v/w) for samples collected from Becher (southwestern Algeria) and Oued Souf Sahara (southeastern Algeria), respectively. Such variability in EO yield can be attributed to multiple factors, including geographical origin, edaphoclimatic conditions, harvest stage, and methodological differences in distillation.

GC–MS analysis identified trans‐thujone (51.9%) as the dominant compound, followed by santolina triene (10.6%), sabinene (6.2%), eucalyptol (5.3%), cis‐verbenyl acetate (5.1%), camphor (2.6%), α‐pinene (2.0%), terpinen‐4‐ol (1.7%), δ‐terpinene (1.6%), and β‐terpineol (1.4%). Together, these major constituents contribute to the distinctive chemical fingerprint of *C. cinerea* EO. Minor components such as α‐thujene, camphene, p‐cymene, and carvotanacetone were also detected in lower amounts, further reflecting the oil's chemical diversity [50, 51, 52].

A summary of the tentatively identified compounds, their relative peak‐area percentages, and retention indices (RI) is provided in Table 1. Because no authentic standards, internal standards, or response‐factor corrections were used, the area percentages are semi‐quantitative and should not be interpreted as absolute concentrations. The composition describes the analyzed oil but does not by itself identify the constituents responsible for biological activity.

**TABLE 1:** Tentatively identified constituents of *C. cinerea* essential oil and their semi‐quantitative relative peak‐area percentages from one analytical GC–MS injection (*n* = 1 profile).

Compound	Area (%)	RI (measured)	RI (lit.)
Santolina triene	10.6	908	910
α‐Thujene	0.9	931	932
α‐Pinene	2.0	939	939
Camphene	0.8	953	954
Sabinene	6.2	976	975
β‐Pinene	0.6	980	979
Dehydrocineole	0.6	991	990
β‐Myrcene	tr	991	992
m‐Mentha‐1(7),8‐diene	tr	999	1000
α‐Terpinene	0.8	1018	1017
o‐Cymene	0.4	1022	1021
p‐Cymene	0.6	1026	1025
Eucalyptol	5.3	1033	1032
δ‐Terpinene	1.6	1062	1060
Sabinene hydrate	0.5	1068	1070
Terpinolene	0.4	1088	1085
β‐Thujone	0.5	1102	1100
trans‐Thujone	51.9	1114	1113
Camphor	2.6	1143	1140
β‐Terpineol	1.4	1163	1160
Terpin‐4‐ol	1.7	1177	1175
α‐Terpineol	0.6	1189	1187
Myrtenol	0.1	1194	1192
Neo‐dihydrocarveol	0.5	1226	1225
Carvotanacetone	0.9	1246	1245
cis‐Verbenyl acetate	5.1	1262	1260
Isopulegyl acetate	tr	1273	1270
p‐Cymen‐7‐ol	tr	1287	1285
Neryl acetate	tr	1365	1360
Jasminaldehyde	0.1	1394	1390
Germacrene D	tr	1480	1478
Total	**96.0**	—	—

*Note*: **RI** (measured) is the retention index determined experimentally using a homologous series of n‐alkanes under the same chromatographic conditions. **RI** (lit) refers to the retention index reported in the literature for the same compound.

Abbreviations: RI, retention index; tr, trace.

### 3.2 In Vivo Evaluation of Anti‐Inflammatory Activity

#### 3.2.1 Assessment of Organ Weight Index

Rats treated with benzylthiouracil (BTU) exhibited a marked increase in liver and kidney weight indices (4.36% ± 0.18% and 0.65% ± 0.01%, respectively), reflecting organ hypertrophy and inflammatory damage associated with BTU‐induced toxicity. In contrast, administration of *C. cinerea* EO (BTU + CC group) significantly attenuated these alterations, restoring liver and kidney indices to 2.7% ± 0.25% and 0.52% ± 0.005%, respectively (Figure 1). These values were comparable to those of the ibuprofen‐treated group, suggesting that *C. cinerea* EO exerts a protective effect against BTU‐induced organ dysfunction. Such findings are consistent with previous reports highlighting the hepatoprotective and anti‐inflammatory potential of *C. cinerea* in vivo [53].

**FIGURE 1:**
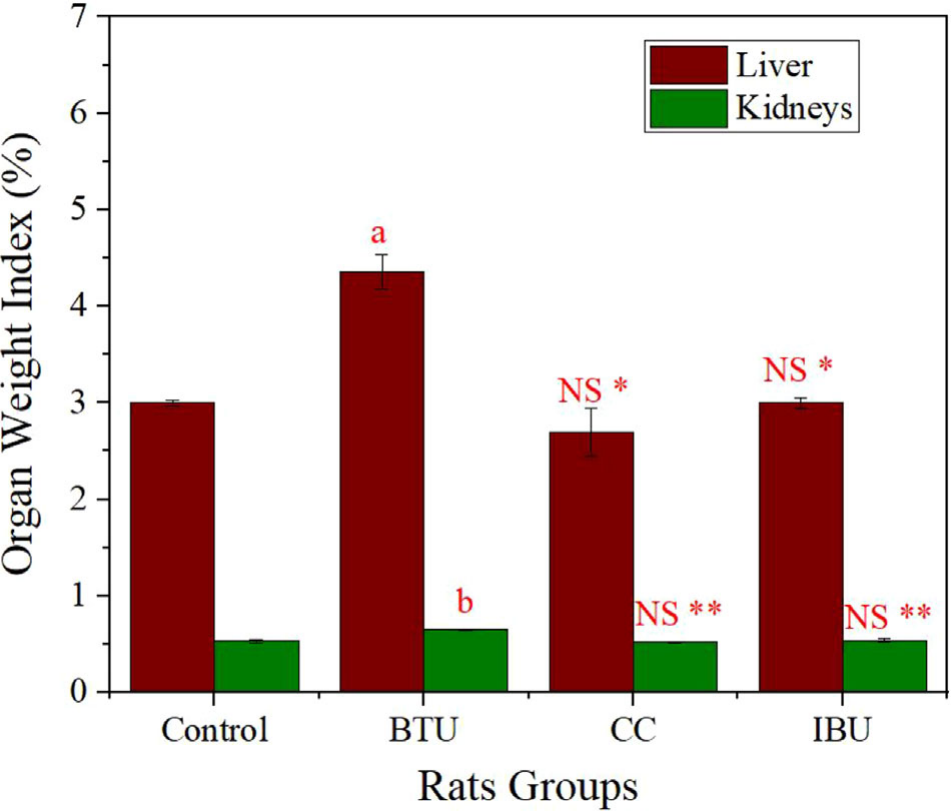
Organ‐weight indices in the four experimental groups. Values are mean ± SD (*n* = 5 independent rats per group). Ordinary one‐way ANOVA followed by Tukey's test; two‐sided *α* = 0.05. Superscripts a, b, and c denote *p* < 0.05, *p* < 0.01, and *p* < 0.001 versus control; *, **, and *** denote the same thresholds versus BTU. BTU, benzylthiouracil; C, control; CC, *Cotula cinerea* essential oil; IBU, ibuprofen.

#### 3.2.2 Analysis of Hematological Parameters

Exposure to BTU caused profound hematological disturbances, including a significant rise in total white blood cell (WBC) and lymphocyte (LYM) counts, alongside a notable decline in hemoglobin (HGB) and red blood cell (RBC) levels. These changes are indicative of systemic inflammation and anemia commonly associated with thiouracil‐induced toxicity [54].

As shown in Table 2, treatment with *C. cinerea* EO effectively restored these parameters toward control values. WBC and LYM counts were reduced to near‐normal levels, while HGB and RBC were significantly improved, indicating a reversal of BTU‐induced anemia and leukocytosis. Platelet counts (PLT), which were elevated under BTU exposure, were also normalized upon *C. cinerea* treatment, further supporting its hematoprotective and anti‐inflammatory role.

**TABLE 2:** Hematological parameters in the four experimental groups (mean ± SD; *n* = 5 rats per group).

	WBC (×10^9^/L)	LYM (×10^9^/L)	GRA (×10^9^/L)	HGB (g/dL)	RBC (×10^12^/L)	PLT (×10^9^/L)
Control	1.46 ± 0.29	1.40 ± 0.26	1.10 ± 0.40	14.46 ± 0.78	7.80 ± 0.47	516.0 ± 9.23
BTU	4.23 ± 0.18^c^	3.10 ± 0.08^c^	0.16 ± 0.08^b^	9.40 ± 0.21^c^	4.90 ± 0.14^c^	676.0 ± 11.50^c^
BTU + CC	2.63 ± 0.31^b***^	2.23 ± 0.12^c***^	1.70 ± 0.49^a***^	15.40 ± 0.12^a***^	7.17 ± 0.11^b***^	456.3 ± 44.0^b***^
BTU + IBU	4.67 ± 0.88^c^	3.00 ± 0.45^c^	1.06 ± 0.14^**^	15.16 ± 0.20^***^	6.30 ± 0.19^c***^	761.3 ± 18.0^c***^

*Note*: Ordinary one‐way ANOVA followed by Tukey's test; two‐sided *α* = 0.05. Superscripts a, b, and c denote *p* < 0.05, *p* < 0.01, and *p* < 0.001 versus control; *, **, and *** denote the same thresholds versus BTU. Absence of a marker denotes *p* ≥ 0.05.

The ibuprofen‐treated group showed partial recovery of several hematological parameters, whereas the EO‐treated group showed values closer to control for some endpoints (Table 2). These between‐group patterns should be interpreted endpoint by endpoint and do not establish superiority of the EO over ibuprofen.

#### 3.2.3 Determination of Glycemic Levels, Hepatorenal Function Markers

Administration of benzylthiouracil (BTU) led to profound metabolic and organ‐related alterations. Fasting blood glucose (FBS) increased markedly, reflecting a state of hyperglycemia, while liver enzymes (AST and ALT) were significantly elevated, indicating hepatocellular damage. In parallel, serum urea levels rose, suggesting renal dysfunction likely associated with BTU‐induced oxidative stress and impaired clearance. These observations are consistent with previous studies that have documented BTU‐mediated hepatorenal toxicity and endocrine disruption [53].

Treatment with *C. cinerea* EO (BTU + CC group) was associated with lower fasting blood glucose and lower ALT, AST, and urea values than the BTU group (Table 3), with several measures approaching the control range. These findings indicate improvement within this experimental model but do not establish an antihyperglycemic mechanism.

**TABLE 3:** Glycemia and hepatic and renal function parameters in the four experimental groups (mean ± SD; *n* = 5 rats per group).

	FBS (g/L)	AST (U/L)	ALT (U/L)	Urea (g/L)	CT (mg/L)
Control	0.58 ± 0.04	126.13 ± 6.48	79.23 ± 2.90	0.43 ± 0.02	5.06 ± 0.07
BTU	0.93 ± 0.01^c^	226.06 ± 8.84^c^	116.00 ± 2.63^c^	0.66 ± 0.01^c^	5.56 ± 0.03^b^
BTU + CC	0.76 ± 0.01^c***^	111.10 ± 18.70^***^	71.56 ± 2.17^a***^	0.43 ± 0.02^***^	4.63 ± 0.27^b***^
BTU + IBU	0.63 ± 0.03^a***^	102.30 ± 9.00^a***^	78.00 ± 5.70^***^	0.47 ± 0.02^b***^	4.63 ± 0.24^b***^

*Note*: Ordinary one‐way ANOVA followed by Tukey's test; two‐sided *α* = 0.05. Superscripts a, b, and c denote *p* < 0.05, *p* < 0.01, and *p* < 0.001 versus control; *, **, and *** denote the same thresholds versus BTU. Absence of a marker denotes *p* ≥ 0.05.

The ibuprofen‐treated group also showed improvement in liver and kidney markers. Differences between the EO and ibuprofen groups varied by endpoint; therefore, the data support comparative activity in this model rather than a general claim that either treatment has a broader protective spectrum.

#### 3.2.4 Quantification of C‐Reactive Protein (CRP)

Benzylthiouracil (BTU) administration caused a pronounced increase in plasma C‐reactive protein (CRP) levels (21 ± 0.57 mg/L), reflecting systemic inflammation and an acute‐phase response (Figure 2). This finding is consistent with earlier studies demonstrating that BTU stimulates pro‐inflammatory cytokine release and induces oxidative tissue stress, both of which contribute to elevated CRP synthesis [55, 56].

**FIGURE 2:**
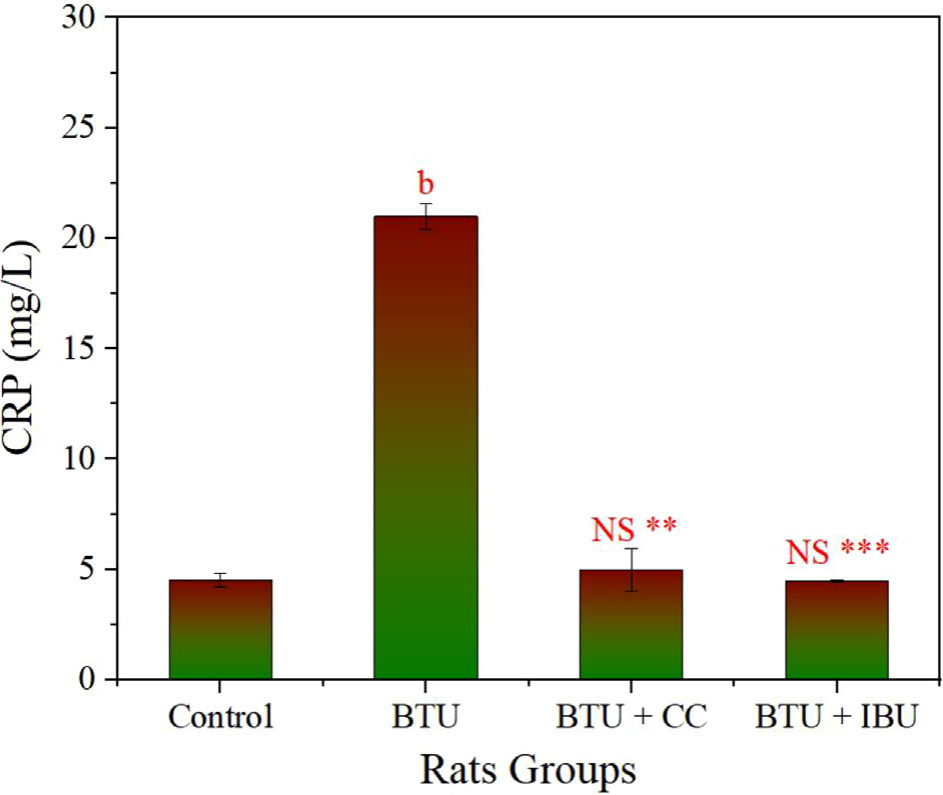
Plasma C‐reactive protein concentrations in the four experimental groups. Values are mean ± SD (*n* = 5 independent rats per group). Ordinary one‐way ANOVA followed by Tukey's test; two‐sided *α* = 0.05. Superscripts a, b, and c denote *p* < 0.05, *p* < 0.01, and *p* < 0.001 versus control; *, **, and *** denote the same thresholds versus BTU. BTU, benzylthiouracil; C, control; CC, *Cotula cinerea* essential oil; IBU, ibuprofen.

Treatment with *C. cinerea* EO was associated with lower CRP concentrations than the BTU group, with values moving toward those of the control group. Ibuprofen produced a similar direction of change. These data support an anti‐inflammatory effect in the BTU model, but safety, dose response, and clinical equivalence to a nonsteroidal anti‐inflammatory drug were not assessed.

#### 3.2.5 Histopathological Examination

##### 3.2.5.1 Liver Histopathology

Histological examination of liver tissues revealed well‐preserved hepatic architecture in the control group, with normal hepatocyte cords and no signs of inflammatory infiltration. In contrast, the BTU‐treated group showed severe hepatic damage characterized by hepatocellular degeneration, disruption of hepatic cords, and dense inflammatory cell infiltration, confirming BTU‐induced hepatotoxicity. These alterations are consistent with previous studies reporting hepatic inflammation and oxidative injury following benzylthiouracil exposure [56].

As shown in Figure 3, ibuprofen treatment (BTU + IBU) reduced inflammatory infiltration, but moderate architectural disorganization remained. The BTU + CC sections showed reduced inflammation and improved cellular integrity relative to BTU alone. Because individual constituents and constituent combinations were not tested, these tissue changes cannot be attributed to a specific molecule or to synergy within the EO.

**FIGURE 3:**
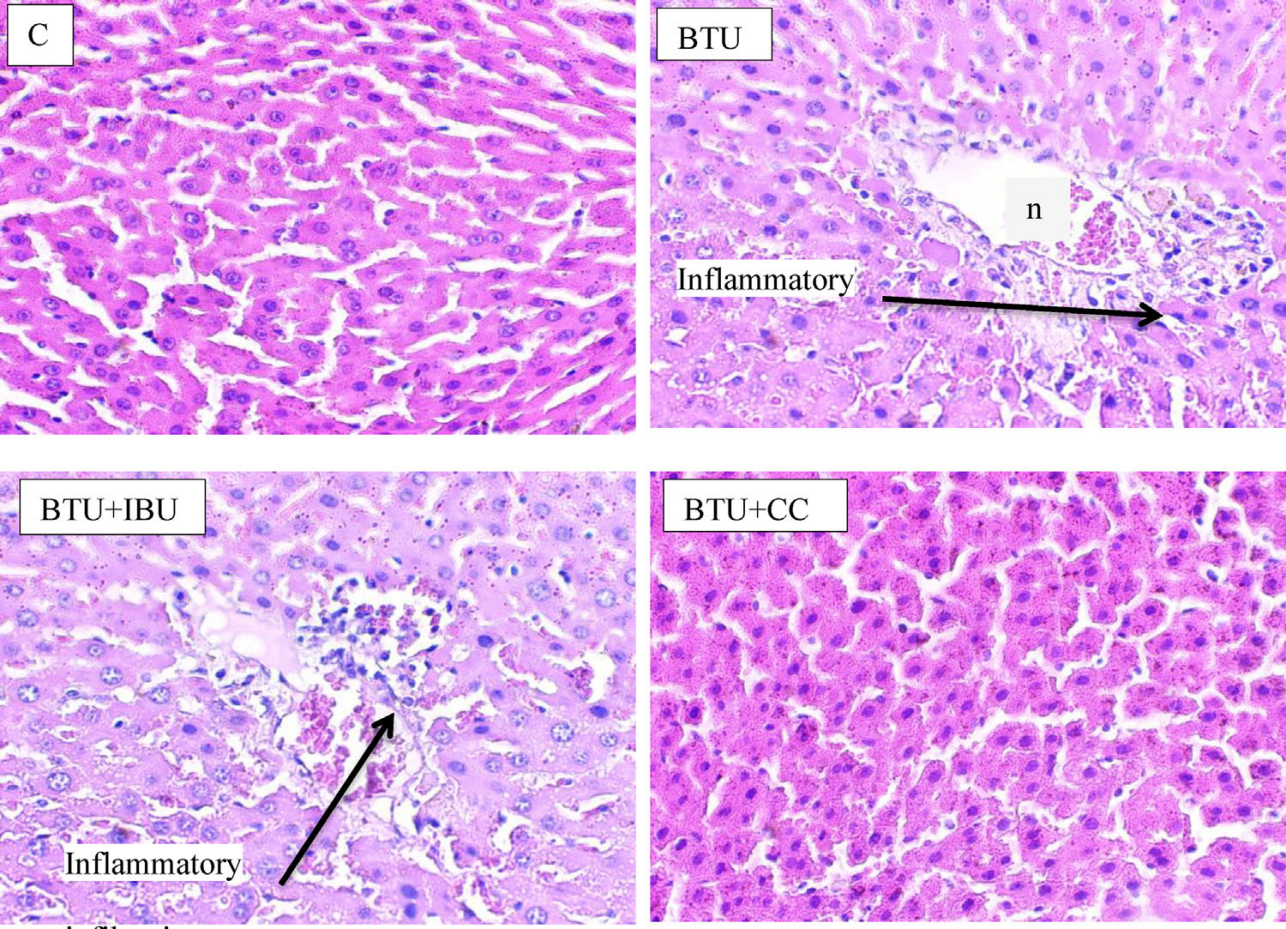
Representative microscopic liver sections from *n* = 5 rats per group: BTU, benzylthiouracil; BTU + IBU, ibuprofen‐treated; BTU + CC, *Cotula cinerea* essential‐oil‐treated; C, control. Magnification ×40. No inferential test was applied to the representative images.

##### 3.2.5.2 Kidney Histopathological

Kidney sections from the control group displayed intact glomeruli and well‐preserved renal tubules, indicating normal histological features. In stark contrast, the BTU group revealed extensive nephrotoxicity, including tubular necrosis, glomerular damage, inflammatory cell infiltration, and overall disruption of renal architecture. These changes corroborate earlier reports that link BTU exposure to oxidative stress–mediated nephrotoxicity and immune activation [53, 57].

Ibuprofen treatment (BTU + IBU) markedly attenuated these pathological changes, with clear signs of renal tissue recovery and reduced inflammation, confirming its nephroprotective efficacy. Meanwhile, *Cotula cinerea* treatment (BTU + CC) provided moderate but notable improvement: partial restoration of glomerular and tubular integrity, accompanied by a visible decline in immune cell infiltration (Figure 4). Although less pronounced than ibuprofen, this nephroprotective effect aligns with previous findings attributing antioxidant and anti‐inflammatory activities to *C. cinerea* EO in renal tissues [53, 58].

**FIGURE 4:**
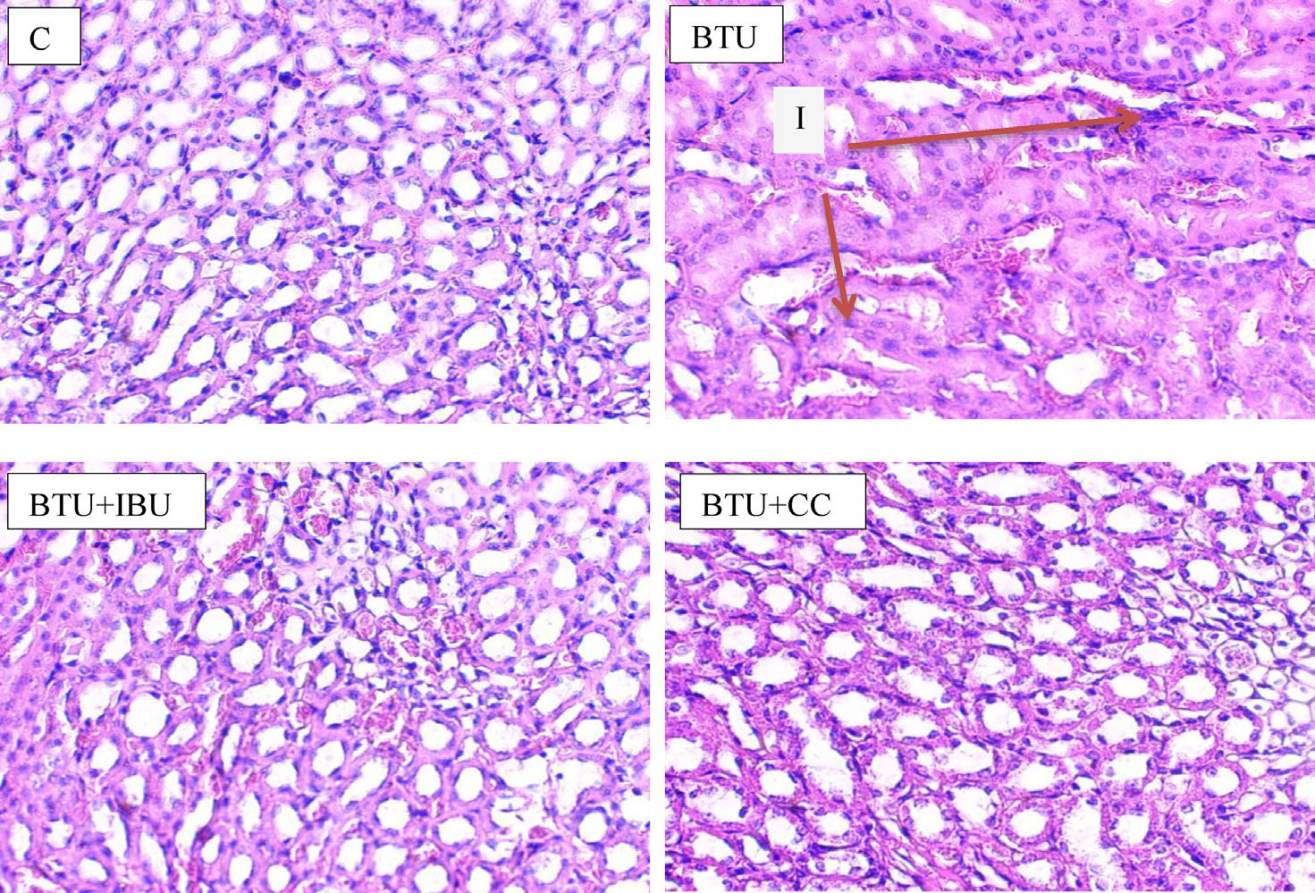
Representative microscopic kidney sections from *n* = 5 rats per group: C, control; BTU, benzylthiouracil; BTU + IBU, ibuprofen‐treated; BTU + CC, *Cotula cinerea* essential‐oil‐treated. Magnification ×40. No inferential test was applied to the representative images.

### 3.3 Molecular Docking Analysis

To explore potential molecular interactions, docking simulations were performed for phytoconstituents present at ≥1% relative abundance against cyclooxygenase‐2 (COX‐2; PDB ID 1PXX) and bovine serum albumin (BSA; PDB ID 6QS9). Diclofenac (DIF) was included as a reference compound. Docking scores were interpreted as predicted interaction metrics rather than evidence of biological activity.

The docking protocol was evaluated by redocking the native co‐crystallized ligands into their respective binding sites. Root mean square deviations were 0.991 Å for COX‐2 and 1.778 Å for BSA, both below the prespecified 2.0 Å threshold (Figure 5; Table 4). These values support pose reproduction within the computational workflow but do not validate biological activity.

**FIGURE 5:**
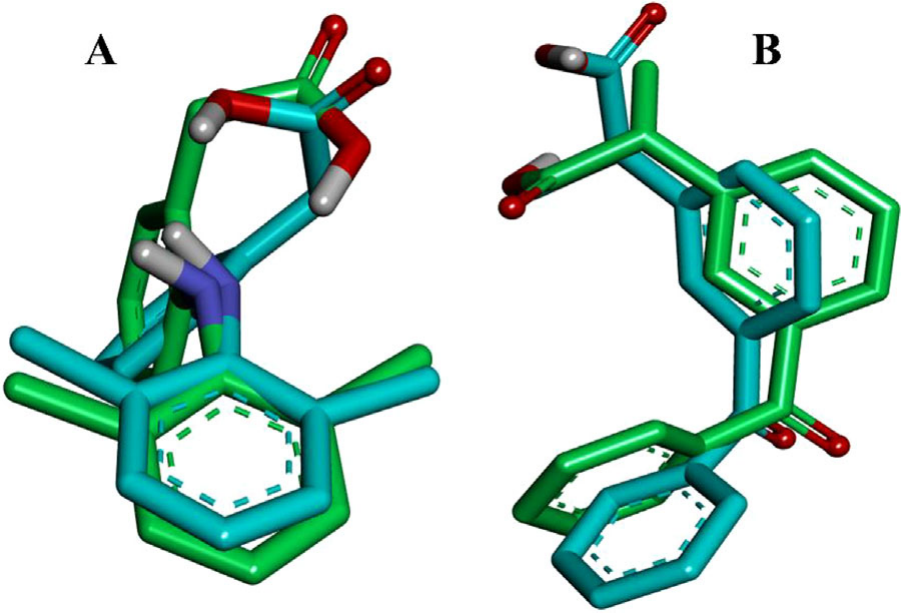
Superimposed docking poses showing the native ligand (green) and redocked conformation (blue) in the active sites of COX‐2 (A) and BSA (B).

**TABLE 4:** Predicted docking scores (kcal/mol) of *C. cinerea* phytochemicals and the reference drug against COX‐2 (1PXX) and BSA (6QS9).

	1PXX	6QS9
RMSD Å	0.991	1.778
Native ligand	−7.99	−8.05
DIF	−7.91	−7.07
CVA	−7.01	−6.93
CAM	−6.66	−6.37
T4O	−6.76	−6.22
EUC	−6.17	−5.69
APN	−6.18	−5.89
DTN	−6.31	−5.32
TTH	−6.11	−5.67
SAN	−5.39	−4.76
BTO	−6.61	−5.5
SAB	−5.97	−5.15

Several phytochemicals produced favorable predicted docking scores for COX‐2 and BSA, although the values cannot establish activity or selectivity. Cis‐verbenyl acetate (CVA) gave the most favorable score among the tested EO constituents for COX‐2 (−7.01 kcal/mol), compared with −7.99 kcal/mol for the native ligand and −7.91 kcal/mol for diclofenac. CVA also produced a predicted BSA score of −6.93 kcal/mol.

Camphor (CAM) and terpin‐4‐ol (T4O) produced predicted COX‐2 scores of −6.66 and −6.76 kcal/mol, respectively, and BSA scores of −6.37 and −6.22 kcal/mol. These values suggest possible interactions that require experimental confirmation.

Other terpenoids, including β‐terpineol, eucalyptol, α‐pinene, δ‐terpinene, and trans‐thujone, produced moderate predicted COX‐2 scores. Their weaker predicted BSA scores describe the docking model only and should not be used to infer systemic distribution.

Sabinene and santolina triene produced the least favorable docking scores among the tested constituents for both proteins. These relative rankings do not exclude biological contributions through other targets or mixture‐level effects.

Within the computational model, CVA produced the most favorable scores among the EO constituents, while CAM and T4O also showed predicted interactions. Because fractionation, standardized testing of individual compounds, and mixture dose–response experiments were not performed, the results do not demonstrate constituent‐specific activity, additivity, synergy, or a quantitative structure–activity relationship.

For COX‐2 (1PXX), the native ligand formed three hydrogen bonds (Arg89, Tyr324, and Val492) and hydrophobic contacts with Val318, Leu321, Leu353, Tyr354, Trp356, Phe487, Met491, Gly495, Ala496, Ser499, and Leu500 (Figure S1). Diclofenac formed two hydrogen bonds (Arg89 and Tyr324), CAM and CVA each formed one hydrogen bond with Ala496, and T4O formed four hydrogen bonds involving Met491, Val492, and Gly495 (Figure 6). These modeled contacts require experimental validation.

**FIGURE 6:**
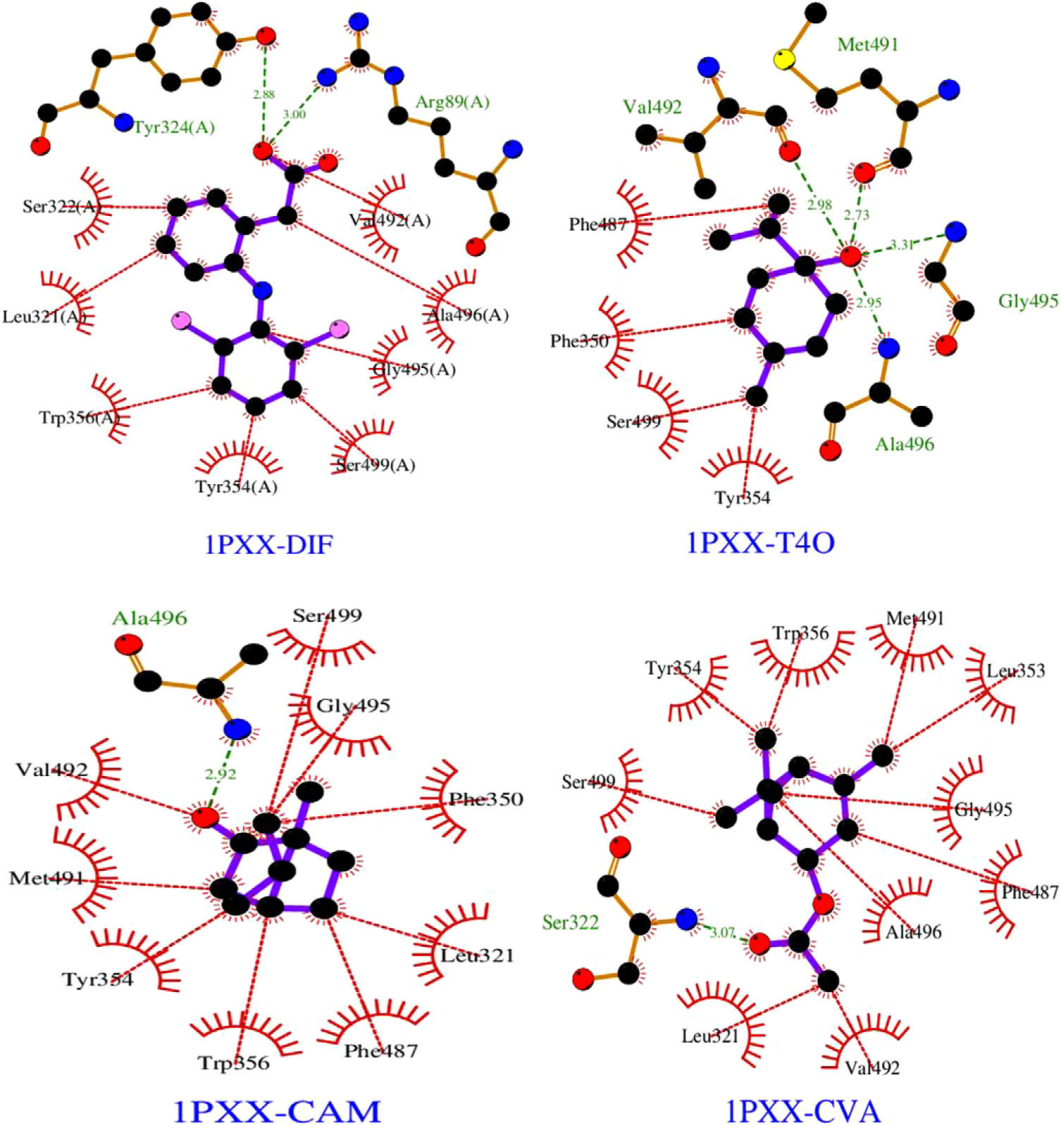
Predicted binding interactions of CAM, CVA, and DIF within the active site of COX‐2.

Within BSA (6QS9), the native ligand was stabilized by four hydrogen bonds (Tyr146, Arg214, Arg253, and Ala287) and hydrophobic contacts (Figure S1). Diclofenac formed three modeled hydrogen bonds involving Arg253 and Ser283; CAM and CVA each formed one with Arg253, and T4O formed two involving Arg253 and Ser283 (Figure 7). These contacts describe the docking poses only.

**FIGURE 7:**
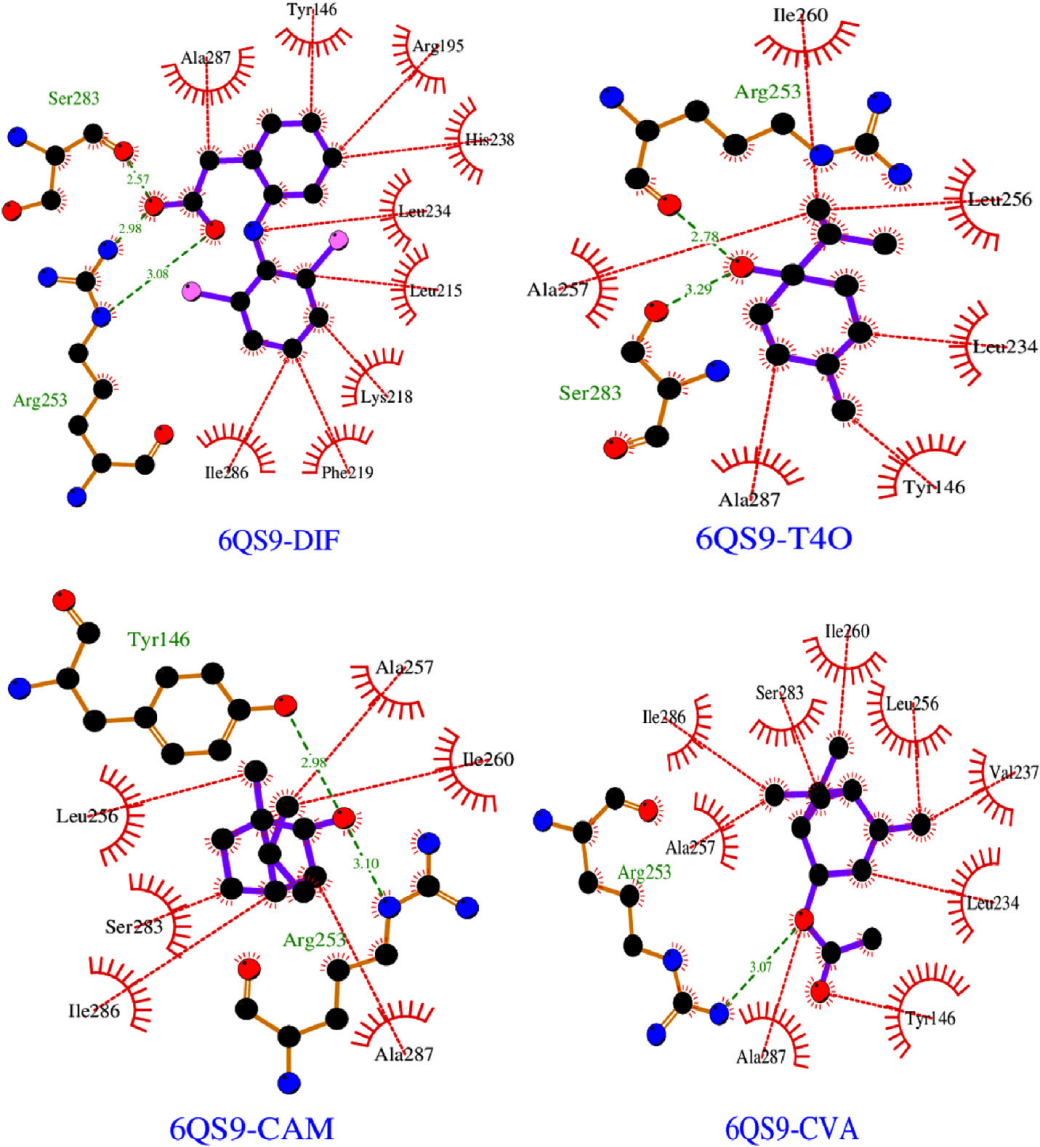
Predicted binding interactions of CAM, CVA, and DIF within the binding site of BSA.

### 3.4 ADMET and Drug‐Likeness Prediction

To complement docking analysis, ADMET profiling was performed for CVA, CAM, and T4O in comparison with diclofenac (DIF), focusing on absorption, distribution, metabolism, excretion, toxicity, and drug‐likeness filters (Table 5).

**TABLE 5:** Predicted ADMET and drug‐likeness properties of the *C. cinerea* lead phytochemicals CVA, CAM, and T4O compared with diclofenac (DIF).

	DIF	CVA	CAM	T4O
Log S (ESOL)	−4.76	−3.26	−2.16	−1.81
Caco‐2 (log *P*app in 10^−6^ cm/s)	1.225	1.618	3.555	1.598
GI absorption (%)	77.74	97.16	95.97	1.60
P‐gp substrate	No	Yes	No	No
VDss (log L/kg)	−1.412	0.35	0.331	0.192
CYP1A2 inhibitor	No	No	NO	No
CYP2C19 inhibitor	No	No	No	No
CYP2C9 inhibitor	No	No	No	No
CYP2D6 inhibitor	No	No	No	No
CYP3A4 inhibitor	No	No	No	No
Clearance (log mL/min/kg)	−0.83	0.251	0.109	1.074
Renal OCT2 substrate	No	No	No	No
Lipinski	Yes (0V)	Yes (0V)	Yes (0V)	Yes (0V)
Ghose	Yes	Yes	No (1V)	Yes
Veber	Yes	Yes	Yes	Yes
Egan	Yes	Yes	Yes	Yes
Muegge	Yes	No (0V)	No (2V)	No (1V)
BAS	0.55	0.55	0.55	0.55
LD50 (mol/kg)	2.162	1.764	1.653	1.949
AMES	No	No	No	Yes
hERG I inhibitor	No	No	No	No
Hepatotoxicity	No	No	No	No
Skin sensitization	No	No	Yes	Yes

#### 3.4.1 Absorption

Predicted solubility and permeability differed among the compounds (Table 5). These values are computational estimates and should be interpreted as prioritization criteria rather than measured formulation or absorption properties.

#### 3.4.2 Distribution

VDss values indicated moderate tissue penetration for CVA (0.35 log L/kg) and CAM (0.331). T4O showed more limited distribution (0.192), while diclofenac exhibited a markedly lower VDss (–1.412), consistent with its tendency to remain in plasma rather than distribute broadly into tissues [59].

#### 3.4.3 Metabolism

The selected prediction tools did not flag inhibition of the listed cytochrome P450 isoforms. This output does not exclude metabolism‐related interactions and requires experimental validation.

#### 3.4.4 Excretion

Clearance predictions revealed that T4O (1.074 log mL/min/kg) had the highest clearance, implying rapid elimination and shorter systemic exposure. In contrast, CVA (0.251) and CAM (0.109) demonstrated lower clearance, favoring longer retention. Diclofenac (–0.83) showed the lowest clearance, consistent with its prolonged pharmacological activity [60]. None of the compounds were identified as renal OCT2 substrates.

#### 3.4.5 Drug‐Likeness

The compounds met several rule‐based filters, with differences across the Ghose and Muegge criteria (Table 5). These filters describe chemical‐property ranges and do not demonstrate oral efficacy.

#### 3.4.6 Toxicity

The in silico models predicted different LD50 values and alerts across compounds. T4O was flagged for possible mutagenicity, and CAM and T4O for possible skin sensitization. These predictions are preliminary and do not replace experimental toxicological assessment.

Taken together, the computational outputs prioritize CVA and CAM for follow‐up testing, while T4O carries less favorable predicted absorption, clearance, and toxicity flags. No claim of safety, superiority to diclofenac, or therapeutic suitability can be made without experimental pharmacokinetic and toxicological data.

### 3.5 MD Simulation Analysis

CVA and CAM were selected for molecular dynamics (MD) simulations with BSA and COX‐2 because of their docking scores and predicted ADMET profiles. The trajectories were analyzed over 100 ns using RMSD, RMSF, radius of gyration (*R*_g_), and solvent‐accessible surface area (SASA) as computational stability descriptors (Table 6; Figure 8).

**TABLE 6:** Mean RMSD, RMSF, *R*_g_, and SASA values from MD simulations of CVA, CAM, and diclofenac with COX‐2 and BSA.

	BSA			1PXX			
	DIF	CVA	CAM	DIF	CVA	CAM
RMSD (nm)	0.239	0.328	0.302	0.273	0.253	0.244
RMSF (nm)	0.150	0.175	0.182	0.145	0.142	0.146
SASA (nm^2^)	294.882	298.395	299.109	261.676	257.388	252.416
*R*_g_ (nm)	2.719	2.768	2.762	2.491	2.479	2.465

Abbreviations: CVA, cis‐verbenyl acetate; CAM, camphor; DIF, diclofenac.

**FIGURE 8:**
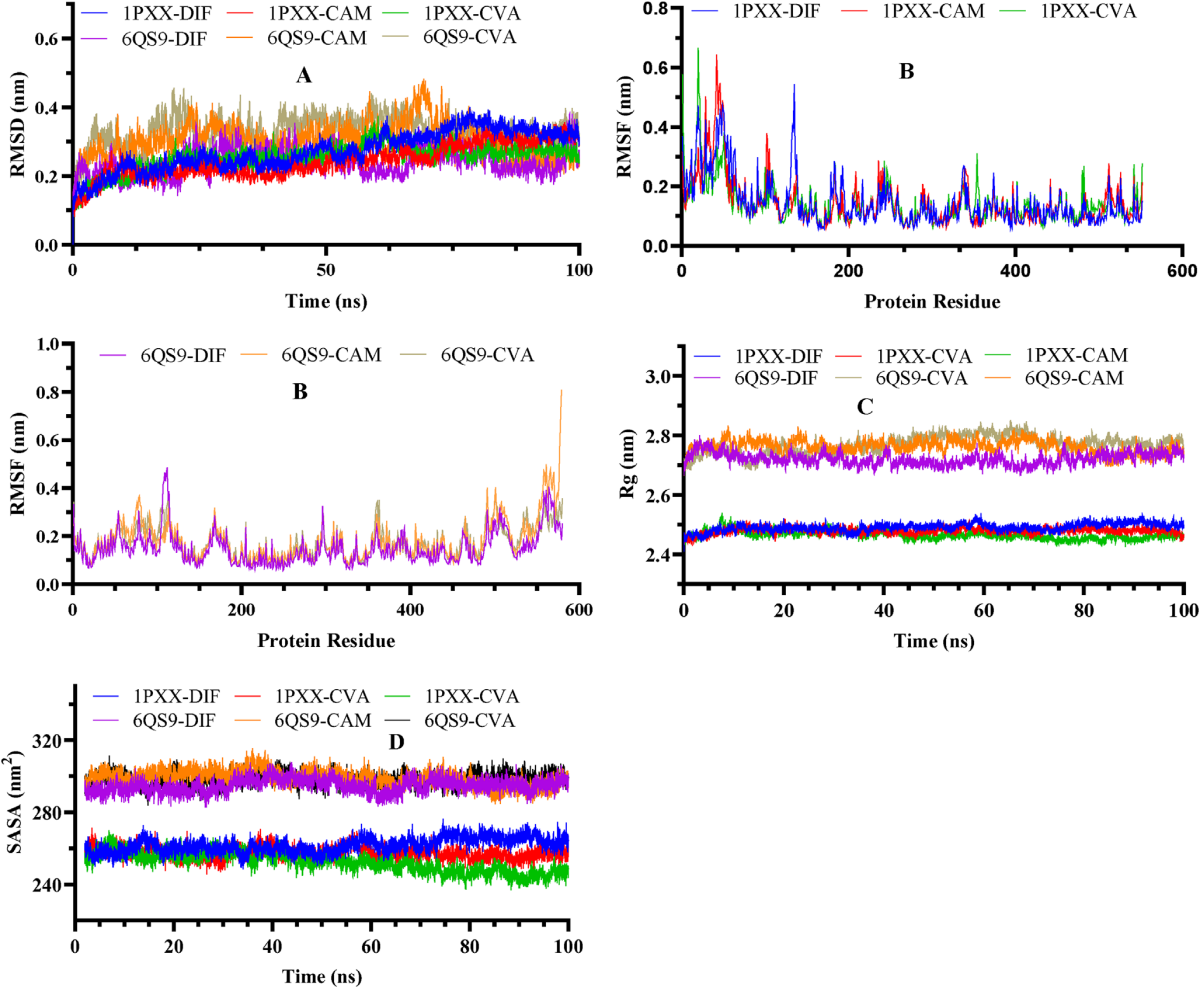
Molecular stability evaluation of COX‐2 and BSA complexes: (A) RMSD; (B) RMSF; (C) *R*_g_; and (D) SASA.

RMSD trajectories for all complexes fluctuated within comparatively narrow ranges during the simulation period (Figure 8A). For BSA, diclofenac showed a lower mean RMSD (0.239 nm) than CVA and CAM, while both phytochemical complexes remained below 0.35 nm. For COX‐2, CVA and CAM showed mean RMSD values of 0.253 and 0.244 nm, respectively, compared with 0.273 nm for diclofenac. These trajectory descriptors do not by themselves establish experimental binding stability.

RMSF profiles showed comparable residue‐level fluctuations among the modeled systems (Figure 8B). In BSA, the larger terminal‐residue fluctuations for CVA and CAM were consistent with flexible terminal regions. COX‐2 complexes showed similar mean RMSF values (approximately 0.14 nm), with localized fluctuations outside the modeled active site.

The radius of gyration (*R*_g_) and SASA analyses further indicated retention of global structural features during the simulations (Figure 8C,D). In BSA complexes, CVA and CAM showed slightly higher *R*_g_ values (2.768 and 2.762 nm) and SASA values (298.395 and 299.109 nm^2^) than diclofenac (*R*_g_, 2.719 nm; SASA, 294.882 nm^2^). In COX‐2 complexes, CVA and CAM showed slightly lower *R*_g_ (2.479 and 2.465 nm) and SASA values (257.388 and 252.416 nm^2^) than diclofenac (*R*_g_, 2.491 nm; SASA, 261.676 nm^2^). These small differences are consistent with limited structural drift in the modeled systems but do not confirm binding, target inhibition, or biological activity.

### 3.6 MM‐PBSA Binding Free Energy Analysis: Results and Discussion

MM‐PBSA calculations were used to estimate binding free energies and energetic contributions for CVA, CAM, and diclofenac in the modeled COX‐2 and BSA complexes (Table S1; Figure 9).

**FIGURE 9:**
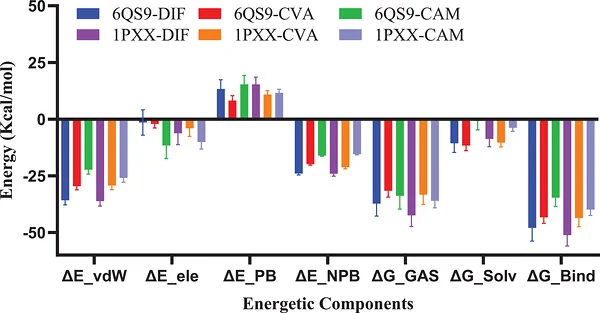
MM‐PBSA energy‐component contributions to modeled ligand binding in BSA and COX‐2 complexes.

For BSA, all ligands exhibited favorable binding free energies, with diclofenac showing the strongest affinity (Δ*G*_Bind_ = −47.97 ± 5.83 kcal/mol), followed by CVA (−43.38 ± 2.64 kcal/mol) and CAM (−34.66 ± 3.86 kcal/mol). Diclofenac binding was primarily driven by van der Waals interactions (Δ*E*_vdW_ = −35.80 ± 2.04 kcal/mol), together with favorable nonpolar solvation (Δ*E*_NPB_ = −23.99 ± 0.64 kcal/mol). CVA displayed moderately strong Δ*E*_vdW_ contributions (−29.57 ± 1.67 kcal/mol) and nonpolar solvation (−19.91 ± 0.48 kcal/mol), resulting in a binding free energy close to diclofenac. In contrast, CAM showed weaker Δ*E*_vdW_ stabilization (−22.31 ± 1.91 kcal/mol); although electrostatic interactions were more favorable (Δ*E*_ele_ = −11.58 ± 5.83 kcal/mol), they were largely offset by an unfavorable polar solvation term (Δ*E*_PB_ = 15.31 ± 4.01 kcal/mol).

For COX‐2, a similar trend was observed, with diclofenac showing the most favorable Δ*G*_Bind_ (−51.20 ± 4.83 kcal/mol), followed by CVA (−43.67 ± 3.80 kcal/mol) and CAM (−39.93 ± 2.59 kcal/mol). Binding was again largely driven by Δ*E*_vdW_ and Δ*E*_NPB_ contributions, particularly for diclofenac (Δ*E*_vdW_ = −36.19 ± 2.20 kcal/mol; Δ*E*_NPB_ = −24.10 ± 1.10 kcal/mol). CVA displayed intermediate stabilization from these terms (Δ*E*_vdW_ = −29.27 ± 1.91 kcal/mol; Δ*E*_NPB_ = −21.21 ± 0.71 kcal/mol), whereas CAM showed weaker Δ*E*_vdW_ (−25.98 ± 1.84 kcal/mol). Although CAM benefited from more favorable Δ*E*_ele_ interactions (−10.09 ± 3.13 kcal/mol), these were counterbalanced by Δ*E*_PB_ (11.64 ± 1.55 kcal/mol).

Overall, the MM‐PBSA estimates suggest that the modeled interactions of CVA and CAM with BSA and COX‐2 were dominated by van der Waals and nonpolar solvation terms. CVA produced more favorable estimated binding energies than CAM but less favorable values than diclofenac. These calculations are comparative predictions and require experimental validation.

### 3.7 Scope and Limitations

This work combines semi‐quantitative chemical profiling, a single BTU‐induced rat model, histology, and computational predictions. The GC–MS assignments were not confirmed with authentic standards, relative peak areas were not corrected for compound‐specific detector responses, and the original TIC is provided in the Supporting Information as Figure S2. A previously included protein‐denaturation dataset was removed during revision because source reconciliation showed that the tabulated concentrations and IC50 values belonged to an α‐amylase assay and could not support a BSA‐denaturation claim. Individual constituents, fractions, and defined mixtures were not tested experimentally, so constituent‐specific activity, synergy, and quantitative structure–activity relationships cannot be inferred. The docking, ADMET, MD, and MM‐PBSA results remain hypothetical until validated by direct binding or enzyme assays. Additional validated in vitro dose‐response, pharmacokinetic, toxicological, formulation, and independent in vivo studies are required.

## 4 Conclusion

The experimental results showed that *C. cinerea* EO was associated with improved inflammatory, hematological, biochemical, and histological endpoints in BTU‐treated rats. These findings support activity in this exploratory model but do not prove a constituent‐specific mechanism.

Computational analyses provided hypothesis‐generating information. CVA and CAM produced favorable predicted interactions with COX‐2 and BSA, and their simulated complexes showed limited structural drift during the analyzed trajectories. ADMET and MM‐PBSA outputs remain predictive and do not establish efficacy, safety, target inhibition, or mechanism.

Taken together, this exploratory study supports further investigation of *C. cinerea* EO as a complex anti‐inflammatory mixture. Future work should include authenticated GC–MS confirmation, fractionation and dose–response testing of individual constituents and defined mixtures, direct target assays, expanded in vivo models, pharmacokinetic and toxicological evaluation, and standardized formulation development before therapeutic conclusions are considered.

## Author Contributions

**Habiba Haddad**: conceptualization, methodology, investigation, data curation, writing – original draft. **Mohammed Larbi Benamor**: supervision, project administration, validation, and writing – review and editing. **Yahia Bekkar**: methodology, formal analysis, software, and data curation. **Ouafa Boudebia**: investigation, formal analysis, visualization. **Salah Neghmouche Nacer**: conceptualization, supervision, validation, and writing – review and editing. **Elhafnaoui Lanez**: formal analysis, methodology, writing – review and editing. **Aicha Adaika** and **Taher M. Abdelhameed**: investigation, resources, data curation. **Lazhar Bechki**: validation, visualization, writing – review and editing. **Touhami Lanez**: conceptualization, supervision, funding acquisition, and writing – review and editing. **Stefania Garzoli**: supervision, writing – review and editing.

## Funding

The authors received no specific funding for this work.

## Conflicts of Interest

The authors declare no conflicts of interest concerning the research, authorship, and publication of this article.

## Declaration of Generative AI and AI‐Assisted Technologies in the Writing Process

During preparation of the revised manuscript and the point‐by‐point response, the authors used OpenAI Codex to assist with language editing, organization, and revision drafting. The authors critically reviewed and edited all generated material and take full responsibility for the final content. The tool was not used to generate experimental or computational data.

## Supporting information




**Supporting File 1**: cbdv71793‐sup‐0001‐SuppMat.docx

## Data Availability

The datasets generated and/or analyzed during the current study are available from the corresponding author on reasonable request.

## References

[1] L. Chen , H. Deng , H. Cui , et al., “Inflammatory Responses and Inflammation‐Associated Diseases in Organs,” Oncotarget 9 (2018): 7204–7218, 10.18632/oncotarget.23208. PMC5805548 29467962

[2] R. Medzhitov , “Origin and Physiological Roles of Inflammation,” Nature 454 (2008): 428–435, 10.1038/nature07201. 18650913

[3] F. W. D. Tai and M. E. McAlindon , “Non‐Steroidal Anti‐Inflammatory Drugs and the Gastrointestinal Tract,” Clinical Medicine 21 (2021): 131–134, 10.7861/clinmed.2021-0039. PMC8002800 33762373

[4] S. Wongrakpanich , A. Wongrakpanich , K. Melhado , and J. Rangaswami , “A Comprehensive Review of Non‐Steroidal Anti‐Inflammatory Drug Use in the Elderly,” Aging and Disease 9 (2018): 143, 10.14336/AD.2017.0306. PMC5772852 29392089

[5] A. Nisar , S. Jagtap , S. Vyavahare , et al., “Phytochemicals in the Treatment of Inflammation‐Associated Diseases: The Journey From Preclinical Trials to Clinical Practice,” Frontiers in Pharmacology 14 (2023): 1177050, 10.3389/fphar.2023.1177050. PMC10203425 37229273

[6] M. G. Miguel , “Antioxidant and Anti‐Inflammatory Activities of Essential Oils: A Short Review,” Molecules 15 (2010): 9252–9287, 10.3390/molecules15129252. PMC6259136 21160452

[7] G. Özkan , F. B. Sakarya , B. Yurt , E. Sieniawska , Y. Yapar , and E. C. Güven , “Bioactive Content and Antioxidant Capacity of Some Plants and Fruits Grown in Türkiye,” Turkish Journal of Agriculture and Forestry 49 (2025): 205–214, 10.55730/1300-011X.3259.

[8] A. Abozeed , “Phytochemical Studies on *Cotula cinerea* L. At Different HABITATS,” Egyptian Journal of Desert Research 63 (2013): 39–54, 10.21608/ejdr.2013.5836.

[9] M. Refaey , M. Abouelela , E. El‐Shoura , et al., “In Vitro Anti‐Inflammatory Activity of *Cotula anthemoides* Essential Oil and In Silico Molecular Docking of Its Bioactives,” Molecules 27 (2022): 1994, 10.3390/molecules27061994. PMC8953163 35335356

[10] M. Lakhdar , “Traditional Uses, Phytochemistry and Biological Activities of *Cotula cinere*a Del: A Review,” Tropical Journal of Pharmaceutical Research 17 (2018): 365, 10.4314/tjpr.v17i2.24.

[11] N. E. Mekhadmi , A. Bentahar , R. Mlik , et al., “Chemical Composition, Anti‐Diabetic, Antioxidant, and Anti‐Inflammatory Potentials of *Cotula cinerea* Growing in Algerian Sahara,” Chemical Papers 78 (2024): 7729–7746, 10.1007/s11696-024-03585-1.

[12] T. Teshima , R. Funai , T. Nakazawa , et al., “ *Coprinopsis cinerea* Dioxygenase Is an Oxygenase Forming 10(S)‐Hydroperoxide of Linoleic Acid, Essential for Mushroom Alcohol, 1‐Octen‐3‐ol, Synthesis,” Journal of Biological Chemistry 298 (2022): 102507, 10.1016/j.jbc.2022.102507. PMC9579011 36122804

[13] N. Mukhopadhyay , A. Shukla , P. N. Makhal , and V. R. Kaki , “Natural Product‐Driven Dual COX‐LOX Inhibitors: Overview of Recent Studies on the Development of Novel Anti‐Inflammatory Agents,” Heliyon 9 (2023): e14569, 10.1016/j.heliyon.2023.e14569. PMC10068128 37020932

[14] J. Liu , C. Peng , Q. Han , et al., “ *Coprinopsis cinerea* Uses Laccase Lcc9 as a Defense Strategy to Eliminate Oxidative Stress During Fungal‐Fungal Interactions,” Applied and Environmental Microbiology 88 (2022): e01760, 10.1128/AEM.01760-21. PMC8752157 34669425

[15] A. Barkat , F. Bouaicha , T. Mester , M. Debabeche , and G. Szabó , “Assessment of Spatial Distribution and Temporal Variations of the Phreatic Groundwater Level Using Geostatistical Modelling: The Case of Oued Souf Valley—Southern East of Algeria,” Water (Basel) 14 (2022): 1415, 10.3390/w14091415.

[16] A. Haddad , H. Guerinik , B. Khezzani , and N. Tarai , “Phytosociology and Distributions of Spontaneous Plant Species in The Northern Zone of The Souf Region (Algeria),” Applied Ecology & Environmental Research 22 (2024): 6187–6205, 10.15666/aeer/2206_61876205.

[17] C. L. Schoch , S. Ciufo , M. Domrachev , et al., “NCBI Taxonomy: A Comprehensive Update on Curation, Resources and Tools,” Database (Oxford) 2020 (2020): baaa062, 10.1093/database/baaa062. PMC7408187 32761142

[18] K. A. Olascuaga‐Castillo , O. Castillo‐Medina , M. Villacorta‐Zavaleta , et al., “Extraction of Essential Oils by Hydrodistillation of Four Aromatic Species: Conditioning, Extraction Conditions, Yield and Chemical Composition,” Scientia Agropecuaria 15 (2024): 385–408, 10.17268/sci.agropecu.2024.029.

[19] M. Samadi , Z. Z. Abidin , H. Yoshida , R. Yunus , and D. R. A. Biak , “Towards Higher Oil Yield and Quality of Essential Oil Extracted From Aquilaria Malaccensis Wood via the Subcritical Technique,” Molecules 25 (2020): 3872, 10.3390/molecules25173872. PMC7503260 32858782

[20] R. Adams , Identification of Essential Oil Components by Gas Chromatography–Mass Spectrometry (Allured Publishing Corporation, 2007).

[21] B. Samah , E. Lanez , M. L. Benamor , et al., “GC/MS Analysis and Computational Evaluation of C. cinerea EO Constituents for Colorectal Cancer: Molecular Docking, Dynamics, and Quantum Chemical Insights Into Cis‐Verbenyl Acetate,” Computational Biology and Chemistry 120 (2025): 108754, 10.1016/j.compbiolchem.2025.108754. 41172603

[22] A. N. Rowan , “Guide for the Care and Use of Laboratory Animals,” Journal of Medical Primatology 8 (1979): 128–128, 10.1159/000460187.

[23] M. G. Neuman , “Hepatotoxicity: Mechanisms of Liver Injury,” in Liver Diseases: A Multidisciplinary Textbook, edited by F. Radu‐Ionita , N. T. Pyrsopoulos , M. Jinga , I. C. Tintoiu , Z. Sun , and E. Bontas (Springer International Publishing, 2020), 75–84, 10.1007/978-3-030-24432-3_7.

[24] R. J. Andrade , G. P. Aithal , E. S. Björnsson , et al., “EASL Clinical Practice Guidelines: Drug‐Induced Liver Injury,” Journal of Hepatology 70 (2019): 1222–1261, 10.1016/j.jhep.2019.02.014. 30926241

[25] M. Lailerd , T. W. Linn , N. Lailerd , D. Amornlerdpison , and A. Imsumran , “Assessment of Antidiabetic and Anti‐Inflammatory Activities of *Carissa carandas* Linn Extract: In Vitro and In Vivo Study,” Applied Sciences 13 (2023): 6454, 10.3390/app13116454.

[26] A. H. El‐desoky , R. F. Abdel‐Rahman , O. K. Ahmed , H. S. El‐Beltagi , and M. Hattori , “Anti‐inflammatory and Antioxidant Activities of Naringin Isolated From *Carissa carandas* L.: In Vitro and In Vivo Evidence,” Phytomedicine 42 (2018): 126–134, 10.1016/j.phymed.2018.03.051. 29655678

[27] S. Bensouiki , F. Belaib , M. Sindt , et al., “Evaluation of Anti‐Inflammatory Activity and In Vitro Drug Release of Ibuprofen‐Loaded Nanoparticles Based on Sodium Alginate and Chitosan,” Arabian Journal for Science and Engineering 45 (2020): 7599–7609, 10.1007/s13369-020-04720-2.

[28] A. Daina , O. Michielin , and V. Zoete , “SwissADME: A Free Web Tool to Evaluate Pharmacokinetics, Drug‐Likeness and Medicinal Chemistry Friendliness of Small Molecules,” Scientific Reports 7 (2017): 42717, 10.1038/srep42717. PMC5335600 28256516

[29] D. E. V. Pires , T. L. Blundell , and D. B. Ascher , “pkCSM: Predicting Small‐Molecule Pharmacokinetic and Toxicity Properties Using Graph‐Based Signatures,” Journal of Medicinal Chemistry 58 (2015): 4066–4072, 10.1021/acs.jmedchem.5b00104. PMC4434528 25860834

[30] N. M. O'Boyle , M. Banck , C. A. James , C. Morley , T. Vandermeersch , and G. R. Hutchison , “Open Babel: An Open Chemical Toolbox,” Journal of Cheminformatics 3 (2011): 33, 10.1186/1758-2946-3-33. PMC3198950 21982300

[31] S. Kim , J. Chen , T. Cheng , et al., “PubChem 2023 Update,” Nucleic Acids Research 51 (2023): D1373–D1380, 10.1093/nar/gkac956. PMC9825602 36305812

[32] H. M. Berman , “The Protein Data Bank,” Nucleic Acids Research 28 (2000): 235–242, 10.1093/nar/28.1.235. PMC102472 10592235

[33] T. Lanez , M. Lanez , R. Lanez , E. Lanez , and B. Talbi‐Lanez , “Computational Screening of Novel Nitroimidazole Candidates: Targeting Key Enzymes of Oral Anaerobes for Anti‐Parasitic Potential,” Current Drug Discovery Technologies 22 (2024): E15701638326365. 10.2174/0115701638326365241029080310 39506436

[34] Y. Bekkar , Z. Saada , and A. Benine , “Computational Analysis of N‐Ferrocenylmethyl‐N‐Phenylbenzohydrazide: Molecular Docking and Dynamic Stability With BSA,” Journal of the Algerian Chemical Society 1 (2024): 29–43, https://www.jacs‐dz.org/index.php/jacs/article/view/338.

[35] Y. Bekkar , E. Lanez , T. Lanez , et al., “Combined In Vitro and In Silico Analysis of Ferrocenylmethylaniline Derivatives: Antibacterial Potential, DFT Calculations, and Molecular Dynamics Insights,” Journal of Organometallic Chemistry 1032 (2025): 123618, 10.1016/j.jorganchem.2025.123618.

[36] A. Adaika , Y. Bekkar , S. Youmbai , et al., “Synthesis, Antioxidant, and Antidiabetic Potential of Ferrocenylmethylnucleobase Compounds: In Vitro, In Silico Molecular Docking, DFT Calculation, and Molecular Dynamic Simulations,” Applied Organometallic Chemistry 39 (2025): e7988, 10.1002/aoc.7988.

[37] R. A. Laskowski and M. B. Swindells , “LigPlot+: Multiple Ligand–Protein Interaction Diagrams for Drug Discovery,” Journal of Chemical Information and Modeling 51 (2011): 2778–2786, 10.1021/ci200227u. 21919503

[38] G. M. Morris , R. Huey , W. Lindstrom , et al., “AutoDock4 and AutoDockTools4: Automated Docking With Selective Receptor Flexibility,” Journal of Computational Chemistry 30 (2009): 2785–2791, 10.1002/jcc.21256. PMC2760638 19399780

[39] M. L. B. Amor , E. Lanez , Y. Bekkar , et al., “Exploring the Interactions of Ferrocenylmethylnucleobase Derivatives with BSA and HHb: Insights from Electrochemical, Spectroscopic, ADMET, In Silico Docking, and MD Simulations,” ChemistrySelect 10 (2025): 1–16, 10.1002/slct.202404678.

[40] Y. Bekkar , E. Lanez , T. Lanez , et al., “Antidiabetic Potential of Synthesized Ferrocenylmethylaniline Derivatives: Insights From In Vitro Studies, Molecular Docking, ADMET, DFT Calculations, and Molecular Dynamics Simulation,” Biotechnology and Applied Biochemistry 73 (2025): 198–211, 10.1002/bab.70014. 40534200

[41] D. Van Der Spoel , E. Lindahl , B. Hess , G. Groenhof , A. E. Mark , and H. J. C. Berendsen , “GROMACS: Fast, Flexible, and Free,” Journal of Computational Chemistry 26 (2005): 1701–1718, 10.1002/jcc.20291. 16211538

[42] M. Bugnon , M. Goullieux , U. F. Röhrig , et al., “SwissParam 2023: A Modern Web‐Based Tool for Efficient Small Molecule Parametrization,” Journal of Chemical Information and Modeling 63 (2023): 6469–6475, 10.1021/acs.jcim.3c01053. PMC10649791 37853543

[43] O. Boudebia , M. L. Benamor , L. Bourougaa , et al., “Holistic Investigation of *Cotula cinerea* Essential Oil Against Diabetes: Hypoglycemic Activity, Enzymatic Inhibition, GC‐MS Characterization, ADMET Forecasting, MD Simulations, and DFT Insights,” Journal of Computer‐Aided Molecular Designs 39 (2025): 80, 10.1007/s10822-025-00664-7. 40952536

[44] N. A. Abdul‐Rida , N. H. Youssef , R. H. Allami , Y. Bekker , L. Elhafnaoui , and N. A. Al‐Masoudi , “Novel Piperazine–Pyrimidine Hybrids as Potential Anticancer Agents Against MCF‐7 Breast Carcinoma: Synthesis, Antimicrobial Activity, Antioxidant Evaluation, and Computational Insights,” Journal of Molecular Structure 1358 (2026): 145287, 10.1016/j.molstruc.2026.145287.

[45] N. A. Abdul‐Ridha , A. M. Youssef , L. Elhafnaoui , et al., “Synthesis, In‐Silico Evaluation, ADME Analysis, and Molecular Dynamics Simulation of Novel β‐Lactam Derivatives Based on Diclofenac and Sulindac With Anticancer and Antioxidant Activities,” Chemistry & Biodiversity 22 (2025): e01586, 10.1002/cbdv.202501586. 40812146

[46] R. Kumari , R. Kumar , and A. Lynn , “g_mmpbsa —A GROMACS Tool for High‐Throughput MM‐PBSA Calculations,” Journal of Chemical Information and Modeling 54 (2014): 1951–1962, 10.1021/ci500020m. 24850022

[47] B. Gogoi , P. Chowdhury , N. Goswami , et al., “Identification of Potential Plant‐Based Inhibitor Against Viral Proteases of SARS‐CoV‐2 Through Molecular Docking, MM‐PBSA Binding Energy Calculations and Molecular Dynamics Simulation,” Molecular Diversity 25 (2021): 1963–1977, 10.1007/s11030-021-10211-9. PMC8047602 33856591

[48] M. Djellouli , H. Benmehdi , S. Mammeri , A. Moussaoui , L. Ziane , and N. Hamidi , “Chemical Constituents in the Essential Oil of the Endemic Plant *Cotula cinerea* (Del.) From the Southwest of Algeria, Asian Pac,” Asian Pacific Journal of Tropical Biomedicine 5 (2015): 870–873, 10.1016/j.apjtb.2015.06.007.

[49] N. E. Mekhadmi , R. Mlik , M. Ramdani , et al., “Chemical Composition and Biological Properties of *Cotula cinerea* Essential Oil From Sahara of Algeria,” Biocatalysis and Agricultural Biotechnology 47 (2023): 102613, 10.1016/j.bcab.2023.102613.

[50] Y. Pyo and Y. J. Jung , “Microbial Fermentation and Therapeutic Potential of p‐Cymene: Insights Into,” Biosynthesis and Antimicrobial Bioactivity, Fermentation 10 (2024): 488, 10.3390/fermentation10090488.

[51] F. S. Sharopov , A. Salimov , S. Numonov , et al., “Phytochemical Study on the Essential Oils of Tarragon (*Аrtemisia dracunculus* L.) Growing in Tajikistan and Its Comparison With the Essential Oil of the Species in the Rest of the World,” Natural Products Communications 15 (2020): 193–200, 10.1177/1934578x20977394.

[52] F. M. Abdoul‐Latif , A. Ainane , I. H. Aboubaker , et al., “Chemical Composition of the Essential Oil of *Catha edulis* Forsk From Djibouti and Its Toxicological Investigations In Vivo and In Vitro,” Processes 11 (2023): 1324, 10.3390/pr11051324.

[53] Z. Lakache , M. Sadallah , H. Aliboudhar , H. Hacib , K. Abdelkrim , and H. Tounssi , “In Vivo Wound Healing and In Vitro Anti‐ Antioxidant Activity Evaluation of *Cotula cinerea* ,” Bulletin of Pharmaceutical Sciences Assiut University 47 (2023): 141–150, 10.21608/bfsa.2023.220458.1798.

[54] F. Bensiradj , M. Hignard , R. Nakkash , et al., “Benzylthiouracil‐Induced ANCA‐Associated Vasculitis: A Case Report and Literature Review,” European Journal of Case Reports in Internal Medicine 6 (2019): 001283, 10.12890/2019_001283. PMC6936924 31893199

[55] F. Kan and I. Kucukkurt , “Investigation of the Effect of Boron on Thyroid Functions and Biochemical Parameters in Hypothyroid Induced‐Rats,” Journal of Biochemical and Molecular Toxicology 36 (2022): e23186, 10.1002/jbt.23186. 35924451

[56] E. A. Amra , S. A. A. El Rehim , F. M. Lashein , and H. S. Shoaeb , “Effect of a Bradykinin Potentiating Factor Separated From Honey Bee Venom on Thyroid Gland and Testis in Hypothyroid White Rats,” Journal of Basic and Applied Zoology 83 (2022): 1, 10.1186/s41936-021-00263-6.

[57] S. T. Ajmi , R. Braham , S. Toumi , et al., “Benzylthiouracil‐Induced Glomerulonephritis,” Case Reports in Medicine 2009 (2009): 1–4, 10.1155/2009/687285. PMC2729276 19721711

[58] Z. Lakache , H. Aliboudhar , M. Sadallah , et al., “Wound Healing and Analgesic Effects of *Brocchia cinerea* Essential Oil in Experimental Animals,” Journal of Biological Research—Bollettino Della Società Italiana Di Biologia Sperimentale 96 (2023): 1, 10.4081/jbr.2023.11346.

[59] M. D. Mellaoui , K. Zaki , K. Abbiche , et al., “In Silico Anticancer Activity of Isoxazolidine and Isoxazolines Derivatives: DFT Study, ADMET Prediction, and Molecular Docking,” Journal of Molecular Structure 1308 (2024): 138330.

[60] S. Khamouli , S. Belaidi , M. Ouassaf , T. Lanez , S. Belaaouad , and S. Chtita , “Multi‐Combined 3D‐QSAR, Docking Molecular and ADMET Prediction of 5‐Azaindazole Derivatives as LRRK2 Tyrosine Kinase Inhibitors,” Journal of Biomolecular Structure & Dynamics 40 (2022): 1285–1298. 10.1080/07391102.2020.1824815 32964807

