# Supplementary material for: Anti‐Inflammatory Activity of Cotula cinerea Essential Oil: Integrated Chemical, In Vivo, Histological, and Computational Evaluation
Source: Chem Biodivers. 2026 Oct 6;23(10):e71793. doi: 10.1002/cbdv.71793 (PMC13640041; doi:10.1002/cbdv.71793)
Supplement: Supplementary file 1 [file CBDV-23-e71793-s001.docx]

**Figure S1.** Binding modes of the native ligands in the active sites of COX-1 (1EQG), COX-2 (1PXX), and BSA (6QS9).

**Table S1.** MM-PBSA energy components (kcal/mol) of CVA, CAM and DIF in complex with BSA and COX-2.

|  | **1PXX-DIF** | **1PXX-CAM** | **1PXX-CVA** | **6QS9-DIF** | **6QS9-CAM** | **6QS9-CVA** |
| --- | --- | --- | --- | --- | --- | --- |
| ΔE_vdW_ | -36.19 ± 2.20 | -25.98 ± 1.84 | -29.27 ± 1.91 | -35.80 ± 2.04 | -22.31 ± 1.91 | -29.57 ± 1.67 |
| ΔE_ele_ | -6.26 ± 5.06 | -10.09 ± 3.13 | -4.03 ± 3.63 | -1.53 ± 5.60 | -11.58 ± 5.83 | -2.10 ± 1.78 |
| ΔE_PB_ | 15.35 ± 3.16 | 11.64 ± 1.55 | 10.84 ± 1.74 | 13.35 ± 3.99 | 15.31 ± 4.01 | 8.20 ± 2.20 |
| ΔE_NPB_ | -24.10 ± 1.10 | -15.49 ± 0.30 | -21.21 ± 0.71 | -23.99 ± 0.64 | -16.08 ± 0.36 | -19.91 ± 0.48 |
| ΔG_Gas_ | -42.46 ± 4.96 | -36.07 ± 3.08 | -33.30 ± 4.39 | -37.33 ± 5.51 | -33.89 ± 5.82 | -31.66 ± 2.80 |
| ΔG_solv_ | -8.74 ± 3.45 | -3.85 ± 1.53 | -10.37 ± 1.95 | -10.64 ± 4.06 | -0.77 ± 4.03 | -11.71 ± 2.20 |
| ΔG_Bind_ | -51.20 ± 4.83 | -39.93 ± 2.59 | -43.67 ± 3.80 | -47.97 ± 5.83 | -34.66 ± 3.86 | -43.38 ± 2.64 |

DIF: diclofenac; CVA: cis-verbenyl acetate; CAM: camphor*.*

**Figure S2**. Representative GC–MS total ion chromatogram (TIC) of Cotula cinerea essential oil. Annotate at minimum camphor (RI 1143) and cis-verbenyl acetate (RI 1262), and preferably trans-thujone (RI 1114), santolina triene (RI 908), and sabinene (RI 976). A chromatogram from another publication must not be presented as raw evidence for this sample.
